# Theoretical Modeling of Viscosity Monitoring with Vibrating Resonance Energy Transfer for Point-of-Care and Environmental Monitoring Applications

**DOI:** 10.3390/mi10010003

**Published:** 2018-12-21

**Authors:** Gorkem Memisoglu, Burhan Gulbahar, Joseba Zubia, Joel Villatoro

**Affiliations:** 1 Department of Communications Engineering, Escuela de Ingeniería de Bilbao, University of the Basque Country (UPV/EHU), Alda. Urquijo s/n, E-48013 Bilbao, Spain; joseba.zubia@ehu.eus (J.Z.); agustinjoel.villatoro@ehu.eus (J.V.); 2 Department of Electrical and Electronics Engineering, Ozyegin University, 34794 Istanbul, Turkey; burhan.gulbahar@ozyegin.edu.tr; 3 IKERBASQUE–Basque Foundation for Science, E-48011 Bilbao, Spain

**Keywords:** förster resonance energy transfer (FRET), viscosity monitoring, fluidic characterization, microfluidics, point-of-care, environmental monitoring

## Abstract

Förster resonance energy transfer (FRET) between two molecules in nanoscale distances is utilized in significant number of applications including biological and chemical applications, monitoring cellular activities, sensors, wireless communications and recently in nanoscale microfluidic radar design denoted by the vibrating FRET (VFRET) exploiting hybrid resonating graphene membrane and FRET design. In this article, a low hardware complexity and novel microfluidic viscosity monitoring system architecture is presented by exploiting VFRET in a novel microfluidic system design. The donor molecules in a microfluidic channel are acoustically vibrated resulting in VFRET in the case of nearby acceptor molecules detected with their periodic optical emission signals. VFRET does not require complicated hardware by directly utilizing molecular interactions detected with the conventional photodetectors. The proposed viscosity measurement system design is theoretically modeled and numerically simulated while the experimental challenges are discussed. It promises point-of-care and environmental monitoring applications including viscosity characterization of blood or polluted water.

## 1. Introduction

Viscosity is one of the most important characteristics of the fluids with important implications in biomedicine such as early diagnosis of diseases using blood viscosity measurements [1]. Viscosity is affected by various mechanisms including shear rate, temperature and concentration [2]. Viscosity is utilized in point-of-care (POC) measurements of blood coagulation, detection of gases and liquid chromatography [3] and environmental monitoring applications including liquid and air [4,5]. Microfluidic platforms and POC solutions promise several advantages compared with macroscale and bulky monitoring tools such as small volume consumption and disposability [6]. There is a rich set of microfluidic viscosity measurement system designs such as electrical impedance measurements [7], magnetic measurements with magnetorestrictive particles and magneto-elastic sensors [3,8], paper-based microfluidic devices which measure the time taken for the liquid to move a certain fixed distance [2], measuring the switching flow-rate of the fluids [1], laser induced capillary wave measurement [9] and measuring the attenuation of sound waves in the microchannel [10]. However, it is highly challenging to propose a scalable system design which can be exploited with the same fundamental principle in both nanoscale and macroscale dimensions. Besides that, the measurement system design which can be easily applied for in-vivo and remote measurements is an open issue.

In this article, a novel viscosity measurement system method is proposed based on vibrating Förster resonance energy transfer (VFRET) introduced in [11,12,13,14,15,16] by utilizing the acoustic vibration of donors inside a fluid medium and measuring optical emissions from nearby acceptor molecules. The vibration amplitude of donors with acoustic excitation inside the fluid medium depends on both the particle properties and viscosity of the fluid. The proposed theoretical model utilizes nonlinear regression analysis on the collected acceptor emission data to estimate both the position of the micro-particle and the viscosity of the fluid medium. The advantages of the proposed viscosity measurement system design compared with rich examples in the state-of-the-art and the competitive POC or environmental monitoring alternatives [1,2,3,7,8,9,10] are listed as follows:

- Pure mechanical method to get measurements without complicated electronic hardware.
- Nanoscale nature with acousto-optical wireless excitation allowing utilization in challenging applications.
- Energy harvesting properties from optical and acoustic excitations in the neighborhood of the donor-acceptor pairs.
- In-vivo measurement capability for future system designs by collecting the emitted photons remotely with in-body photodetectors and acoustic actuation.
- Flexible and robust system design by tuning the lumped size of the donor/acceptor molecules and the acoustic excitation frequency.
- Scalability for both nanoscale and microscale sensor applications.
- Remote monitoring and wireless data collection capability with optical emissions.

The rest of this paper is organized as follows. In Section 2, a system model for the viscosity measurement is introduced. In Section 3, theoretical modeling of acoustically stimulated VFRET is presented. Then, in Section 4, a mathematical procedure and algorithm for viscosity estimation is designed. Materials to be utilized as donor-acceptor pairs with experimental challenges are discussed in Section 5. Numerical simulations are proposed in Section 6 while the discussions, applications and conclusion are presented in Section 7.

## 2. Viscosity Measurement System Architecture

**Figure 1:**
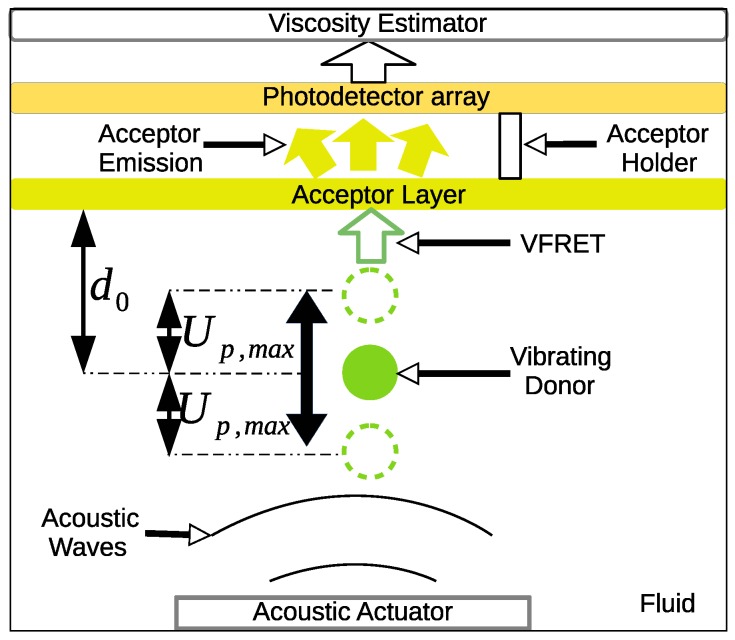
System architecture of the viscosity monitor. Acceptor layer is moved up and down while the donor is vibrated with the amplitude $U_{p,max}$ at the steady state position $d_{0}$ based on the acoustic actuation in the fluid. Measurements are taken for varying $d_{0}$ and collected photons from the acceptor layer are utilized for the viscosity estimation.

**Figure 2:**
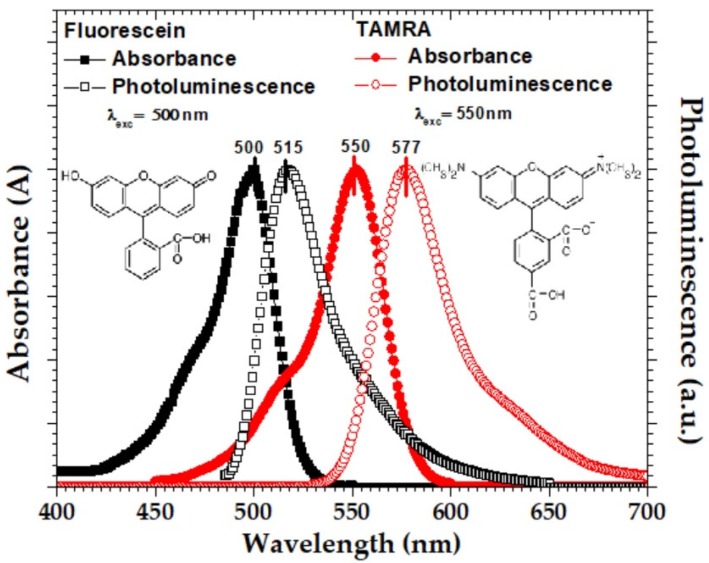
The chemical structures and the normalized absorption and photoluminescence spectra of Fluorescein and 5-Carboxytetramethylrhodamine (TAMRA) materials (solution phase) for the excitation wavelengths at 500 nm and 550 nm, respectively.

The system includes a microfluidic platform consisting of acoustic actuators and photodetectors with the fluid including vibrating and large size spherical particles as lump of the donor molecules as shown in Figure 1. It is assumed that the donor particle reaches the stable or suspended condition under an applied acoustic excitation with the distance $d_{0}$ from the acceptor layer. Furthermore, it is assumed that acceptor layer can move up and down to search for donor molecule changing $d_{0}$. Whenever the acceptor layer is close enough to the donor such as tens to hundreds of nanometer (nm) distances, then the Förster resonance energy transfer (FRET) occurs between the donor particle and the acceptor layer. The emission from the acceptor layer is collected with photodetectors. The donor molecule vibrates with the amplitude of $U_{p,max}$ up and down in a periodic fashion based on the frequency $f_{0}$ of the acoustic excitation. The vibration amplitude and the efficiency of VFRET depend on both the properties of the particle and the fluid. Therefore, the collected emission data is mapped to the viscosity and the particle position estimation by using the non-linear regression analysis as discussed in Section 4. Next, liquid phase optic properties of a donor-acceptor couple (Figure 2) are explained and the dependence of the emission on the fluid parameters are theoretically modeled.

## 3. Theoretical Modeling of VFRET in Fluid

If the fluid is assumed to vibrate with the velocity $v_{f}\left(t\right)={\dot{U}}_{f}\left(t\right)\propto \sin\left(2\;\pi \;f_{0}\;t\right)$ and displacement amplitude $U_{f}\left(t\right)\propto \cos\left(2\;\pi \;f_{0}\;t\right)\;/\;\left(2\;\pi \;f_{0}\right)$, then the maximum displacement amplitude of the fluid denoted by $U_{f,max}$ is given as follows [17]:

(1) $$U_{f,max}=P_{f}\;/\;\left(2\;\pi \;\rho_{f}\;f_{0}\;c_{f}\right)$$

where $P_{f}\left(Pa\right)$ is the acoustic pressure in the fluid, $\rho_{f}\left(\mathrm{kg}\;/\;m^{3}\right)$ is the volume density of the fluid, $c_{f}$ is the sound velocity in the fluid and $f_{0}$ is the acoustic oscillation frequency. If the particle size is assumed to be small and particle diameter is much smaller than the acoustic wavelength, then the oscillation displacement of the particle is represented by the following:

(2) $$U_{p}\left(t\right)=d_{0}-\eta_{p}\;U_{f,max}\;\cos\left(2\;\pi \;f_{0}\;t-\phi_{p}\right)$$

where $U_{p,max}\equiv \eta_{p}\;U_{f,max}$, $d_{0}$ is the suspended or steady-state initial position of the particle, the particle entrainment coefficient $\eta_{p}$ is given by $1\;/\;\sqrt{1\;+\;{\left(\omega_{0}\;\tau_{p}\right)}^{2}}$, the phase difference $\phi_{p}$ equals $\tan^{-1}\left(\omega_{0}\;\tau_{p}\right)$, $\omega_{0}=2\;\pi \;f_{0}$, $\tau_{p}=m_{p}\;/\;\left(3\;\pi \;\mu_{f}\;d_{p}\right)$ is the particle time scale, $m_{p}$ is the particle weight, $\mu_{f}\left(Pa\times s\right)$ is the viscosity of the fluid and $d_{p}$ is the diameter of the particle [17].

The number of photons emitted in the VFRET mechanism for the time duration of Δt at the time *t* is modeled as $N_{FRET}\left(t\right)=\alpha \;E_{FRET}\left(t\right)$ where α is defined as follows [11,14]:

(3) $$\alpha \;\equiv \;\Delta t\;I_{D}\;D_{B}\;\sigma_{D}\Phi_{A}\;N_{D}/\;\left(h_{p}\;f_{D}^{a}\right)$$

where $\Delta t\gg t_{p}$ is the photon counting time, $t_{p}=t_{D}\;+t_{FRET}\;+t_{A}$ is the total time on the order of nanoseconds for photon emissions from the acceptor including the donor excitation time $t_{D}$, FRET time $t_{FRET}$ and the acceptor emission time $t_{A}$, $I_{D}$ is the light intensity in ($W/m^{2}/\mathrm{nm}$) for the donor excitation, $D_{B}$ is the bandwidth of donor excitation in (nm), $f_{D}^{a}=c\;/\;\lambda_{D}^{a}$ is the donor excitation frequency, $c=3\times 10^{8}$ (m/s) is the speed of light, $\lambda_{D}^{a}$ is the donor excitation wavelength, $h_{p}=6.62\times 10^{-34}$ (J·s) is the Planck’s constant, $\sigma_{D}=D_{ext}\;3.825\times 10^{-25}$ is the absorption cross section of donor excitation in (m^2^), $D_{ext}$ is the donor extinction coefficient in M^−1^·cm^−1^ and $\Phi_{A}$ is the quantum yield of acceptors. The dynamic FRET efficiency $E_{FRET}$ oscillating with the vibrating distance between the donor and the acceptor molecules is defined as follows:

(4) $$E_{FRET}\;=\;\frac{k_{A}\;R_{0}^{6}}{d^{6}\;+\;k_{A}\;R_{0}^{6}}$$

where $k_{A}$ is the number of acceptors per donor, *d* and $R_{0}$ are the donor-acceptor and Förster distances, respectively. $\Delta t\ll 1/f_{0}$ is chosen large enough to collect enough number of photons for the measurement process in the receiver photodetectors. The received photon count is modeled under additive white Gaussian assumption for simplicity as follows:

(5) $$N_{FRET}\left(t\right)=\alpha \;E_{FRET}\left(t\right)+n\left(t\right)$$

where n(t) is the noise with the variance $\sigma_{n}^{2}$. Assume that the signal-to-noise ratio (SNR) is defined with respect to the worst case scenario of the noise proportional to the maximum amount of the collected photons as follows:

(6) $$SNR=\frac{\alpha^{2}}{\sigma_{n}^{2}}$$

Then, the final equality representing the relation between the viscosity $\mu_{f}$, $d_{0}$ and the received photon count is defined as follows:

(7) $$N_{FRET}\left(t\right)\;=\frac{\alpha }{1+\frac{1}{k_{A}^{6}\;R_{0}^{6}}{\left(d_{0}-\frac{3\;\mu_{f}\;d_{p}}{\sqrt{9\;\mu_{f}^{2}\;d_{p}^{2}+4\;f_{0}^{2}\;m_{p}^{2}}}\;\frac{P_{f}}{2\;\pi \;\rho_{f}\;f_{0}\;c_{f}}\;\cos\left(2\;\pi \;f_{0}\;t-\tan^{-1}\left(\frac{2f_{0}\;m_{p}}{3\;\mu_{f}\;d_{p}}\right)\right)\right)}^{6}}+n\left(t\right)$$

Next, multiple periods of the received photon count is detected to estimate both $d_{0}$ and $\mu_{f}$.

## 4. Viscosity Estimation Algorithm

**Figure 3:**
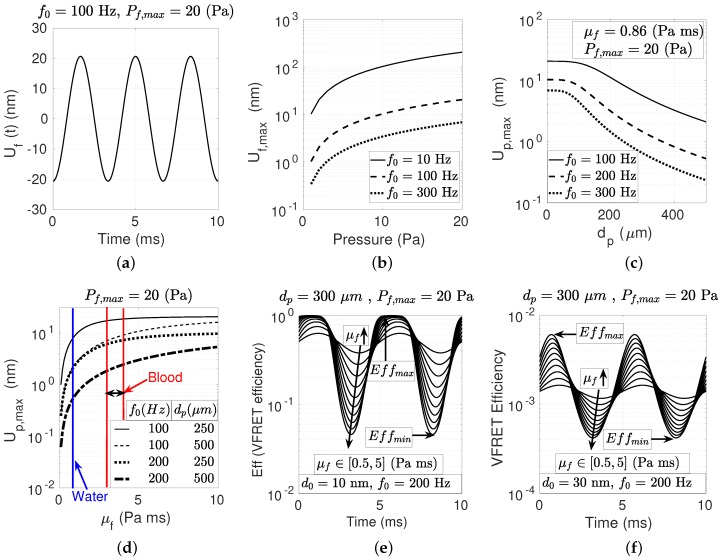
(**a**) The vibration magnitude of the fluid particles for varying time at $f_{0}=100$ Hz and under an acoustic pressure of $P_{f}=20$ Pa and (**b**) its maximum for varying $f_{0}$ and $P_{f}$. (**c**) The maximum vibration amplitude ($U_{p,max}$) of the particle for varying $f_{0}$ and particle diameter $d_{p}$ in water ($\mu_{f}=0.86$ Pa) under $P_{f}=20$ Pa. (**d**) $U_{p,max}$ dependency on $\mu_{f}$ for varying $f_{0}$ and $d_{p}$ with $P_{f}=20$ Pa. (**e**) vibrating Förster resonance energy transfer (VFRET) efficiency for varying $\mu_{f}$ and $d_{0}$ where (**e**) $d_{0}=10$ nm $<U_{p,max}$ and (**f**) $d_{0}=30$ nm $>U_{p,max}$, and $d_{p}=300$ μm, $f_{0}=200$ Hz and $P_{f}=20$ Pa.

A physical search mechanism is applied for getting measurements from the large donor particle for the unknown position. The simple proof-of-concept acceptor sensor is physically moved back and forth to search donor and take vibrating measurements. It is assumed that the measurements corresponding to the form in Figure 3f are utilized since the donor touching the acceptor molecule diminishes the functional information in (7) with constant unit efficiency. The signal processing design for choosing the measurement sets corresponding to $d_{0}>U_{p,max}$ is an open issue. Various nonlinear regression mechanisms and the parameter estimation methods are promising to be utilized to estimate $d_{0}$ and $\mu_{f}$ based on the multiple time measurements with the parametric model in (7) [18]. Multiple periods of the received data is firstly averaged to reduce the effect of the noisy measurement. Assume that $N_{T}$ different periods are utilized, then the mean photon count waveform is given as follows:

(8) $${\bar{N}}_{FRET}\left(t+\tau \right)\;=\frac{1}{N_{T}}\sum_{i=1}^{N_{T}}N_{FRET}\left(t+\tau +\left(i-1\right)T\right)$$

where 0≤τ≤T. Assume that $N_{m}$ different measurements are taken for unknown positions $d_{0}$ while the data points with the FRET efficiency smaller than a pre-defined limit $E_{min}$ are not taken into account, i.e., $E_{FRET}\left(t\right)\geq E_{min}$. In addition, to improve the reliability of the measurements, only the sets with enough number of sample points covering at least $N_{min}$ is taken into account, e.g., more than $N_{min}=T/\left(12\;\Delta t\right)$ samples covering 1/12 of a single period. It is assumed that $d_{0}$ is uniformly distributed between $U_{p,max}\;+\;d_{0,min}\equiv 1$ nm and $U_{p,max}\;+\;d_{0,max}$ which is determined with respect to the maximum amount of displacement magnitude $U_{p,max}$ determined by multiple factors including $\mu_{f}$. For each measurement $i\in [1,N_{m}]$, a nonlinear regression mechanism denoted by NLR(.) is utilized to estimate parameters with respect to the input of ${\bar{N}}_{FRET}\left(t\right)$ between t=0 and *T* with the sample interval of Δt, and the initial estimation of $d_{0,initial}$ and $\mu_{f,initial}$ as follows:

(9) $$\left({\hat{d}}_{0,i},{\hat{\mu }}_{f,i}\right)=NLR\left({\bar{N}}_{FRET}\left(t\right),d_{0,initial},\mu_{f,initial}\right)$$

Then, the final estimation is found with the following by getting average from each measurement:

(10) $${\hat{d}}_{0}=\frac{1}{N_{m}}\sum_{i=1}^{N_{m}}{\hat{d}}_{0,i};\;\;\;{\hat{\mu }}_{f}=\frac{1}{N_{m}}\sum_{i=1}^{N_{m}}{\hat{\mu }}_{f,i};$$

In Monte Carlo simulation studies, MATLAB built-in function nlinfit is utilized as the NLP(.) block with the Levenberg-Marquardt nonlinear least squares algorithm [18].

## 5. Donor-Acceptor Materials and Experimental Challenges

The proposed theoretical modeling allows utilization of different pairs of donor/acceptor pairs while still utilizing the fundamental Equation (7) with different parameters of donors and acceptors. Therefore, it is adaptive with molecular selection capability which is highly important for different mediums requiring specific sets of pairs, e.g., organic or biological mediums with biocompatible molecules compared with environmental monitoring applications. Fluorescent molecules such as dyes, and quantum dots, can be employed in the FRET system as the donor or the acceptor. Fluorescein and 5-TAMRA(5-Carboxytetramethylrhodamine)(TAMRA) are two fluorescent dyes evaluated in this study as donor and acceptor materials, respectively. These materials have various advantages such as great Stokes shifts (difference between excitation and emission wavelength peaks), high spectral overlap area and rich set of applications [19,20,21,22,23]. Figure 2 and Table 1 show the absorption and emission spectra, chemical structures and the specific material properties like high absorption coefficients, quantum yields [19,20,21,22,23]. The spectral overlap between the emission of fluorescein donor fluorophore and the absorption of TAMRA acceptor fluorophore is high as shown in Figure 2. Förster distance $R_{0}$ between the fluorescein donor and the TAMRA acceptor is about 5.5 nm [24]. Both materials have a few nanosecond photoluminescence lifetimes. Both fluorescein and TAMRA are sensitive to the polarity of their surrounding environment [19]. Prevention of self-quenching effects in liquid requires low concentrations (lower than 4 μM for fluorescein, and lower than 250 nM for TAMRA).

There are various experimental challenges to be solved for realizing the proof-of-concept experimental implementation. Donor molecules are assumed to form lumps with a spherical geometry and a large diameter. The method to form lumps with the desired particle size is an open issue. In addition, the interaction of the lumped donor sphere and acceptor layer with the fluid should be modeled and analyzed in detail. If the donor molecule disintegrates, then the speed of disintegration should be measured and the vibration frequency should be optimized with the appropriate measurement time.

## 6. Numerical Simulations

The fluid medium is assumed to be water with the simulation parameters shown in Table 2. Acoustic velocity is assumed to be $c_{f}=1543$ m/s. The range of the viscosity is assumed to be between 0.5 and 5 (Pa ms) covering the range of interest of the water around 0.89 (Pa ms) and blood between 3 and 4 (Pa ms). The donor particle is assumed to be lumped with a large spherical geometry in the diameter range of [1,500] μm. $R_{0}$ is assumed to be 10 nm while $k_{A}=1$ for the worst case performance analysis. $P_{f}$ is varied between 1 and 20 Pa while $f_{0}$ is chosen between 10 and 300 Hz for low frequency excitation with much larger wavelength compared with the particle lump size. $N_{MC}=20$ different simulations are realized and their average are taken to observe the average behavior of the estimates ${\hat{\mu }}_{f}$ and ${\hat{d}}_{0}$. $N_{m}=20$ different measurement sets are taken along $N_{T}=1000$ periods of the received signal for varying $d_{0}$ values distributed uniformly between $\eta_{p}\;U_{f,max}+1$ and $\eta_{p}\;U_{f,max}+20$ nm for each specific setting of $\mu_{f}$ to be estimated with a given SNR. Photon collection interval is set to Δt=20 μs. Minimum efficiency to be included in the estimation algorithm is set to $E_{min}=0.01$ while sample sets with lower than $N_{min}=T/\left(6\;\Delta t\right)$ samples in a single period $T=1/f_{0}$ are discarded since they include mostly noisy samples.

The oscillation magnitude $U_{f}\left(t\right)$ of the fluid for $f_{0}=200$ Hz and $P_{f}=20$ Pa is shown in Figure 3a. The maximum fluid displacement is numerically calculated for varying $f_{0}$ and $P_{f}$ as shown in Figure 3b reaching the ranges of hundreds of nm much larger than $R_{0}$ enough to obtain VFRET. In Figure 3c, it is observed that decreasing the particle lump size $d_{p}$ and $f_{0}$ increases $U_{p,max}$. The effect of $\mu_{f}$ on $U_{p,max}$ is numerically analyzed in Figure 3d for varying $f_{0}$ and $d_{p}$ with $P_{f}=20$ Pa observing the nonlinear dependence of $U_{p,max}$ on $\mu_{f}$ covering the ranges of water and blood for the POC and the environmental monitoring applications. In Figure 3e,f, time domain waveforms of the VFRET efficiencies are shown for varying $\mu_{f}$ and $d_{0}$. If $U_{p,max}>d_{0}$ as shown in Figure 3e, then the information about $\mu_{f}$ dependency is lost due to touching of the donor to the acceptor layer. In Figure 3f, the efficiency does not reach the maximum level of unity due to the larger distance between donor and acceptor which is the case to be utilized in estimating $\mu_{f}$.

**Figure 4:**
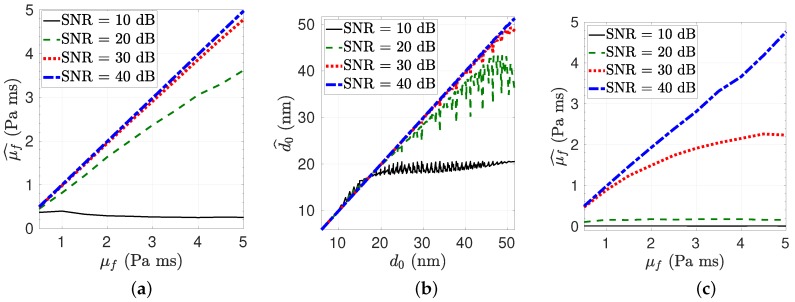
(**a**) The viscosity (${\hat{\mu }}_{f}$) and (**b**) the particle distance (${\hat{d}}_{0}$) estimation performance for varying signal-to-noise ratio (SNR). between 10 dB and 40 dB where $P_{f}=20$ Pa, $d_{p}=500$ μm, $f_{0}=50$ Hz. (**c**) The decrease in estimation performance for varying where $P_{f}=20$ Pa, $d_{p}=300$ μm and $f_{0}=20$ Hz.

The estimation of the viscosity $\mu_{f}$ and donor-acceptor stable distance $d_{0}$ are achieved with the Monte Carlo simulation as shown in Figure 4a,b, respectively, where $P_{f}=20$ Pa, $d_{p}=500$ μm, $f_{0}=50$ Hz and SNR changes between 10 dB and 40 dB. Increasing $\mu_{f}$ or decreasing $d_{p}$ results in an increase in $U_{p,max}$ while decreasing the number of higher efficiency photon collection intervals in a single period. It is observed that the error increases for increased $U_{p,max}$ as shown in Figure 4c where $d_{p}=300$ μm and $f_{0}=20$ Hz. However, there is trade-off such that it is difficult to take measurement from the donor with reduced $U_{p,max}$ since the acceptor needs to be closer to get reliable measurements. If $U_{p,max}$ is larger, then the distance between the donor and acceptor can be large to get a reliable measurement while the number of samples gets lower. On the other hand, the efficiency of the system design requires high SNR which can be improved by tuning the size of the particle, improving photodetector efficiency and the number of measurement intervals. It can also be improved with multiple donor molecules measured in parallel as open issues.

## 7. Applications

The proposed viscosity measurement system is promising for utilization as a low cost micromechanical system in various POC and environmental monitoring applications. The viscosity measurement of the blood with a simple and low cost architecture allows early detection of various cardiovascular or cancer related diseases [6]. Similarly, pollutants in water and oceanic mediums can be analyzed with the proposed viscosity measurement architecture with a POC style. In addition, flow based mechanical interactions of the donor and acceptor molecules with the environment can be converted to VFRET emissions for monitoring complicated fluid mechanics. The adaptive lumped size of the donors and acceptors, and the flexible setting of the acoustic excitation frequency make the proposed system capable of adaptation to different applications.

There are significant advantages of the proposed system due to the low hardware complexity relying on a completely mechanical set-up without requiring any electronic circuitry inside the medium to be monitored. In addition, the collection of the optical signals remotely allows for wireless applications and remote detection of the viscosity in challenging environments. These environments could include an in-vivo medium or energy scarce applications. Monitoring energy is harvested from the remote acoustic vibration and optical excitation. Besides that, adaptive formation of lumped spherical donor molecules provides robustness to different environments. The challenging future environmental applications include the analysis of deep water with tuned size of donor and acceptor molecules allowing measurable VFRET by scanning a set of acoustic frequencies.

## 8. Open Issues and Discussion

There are various open issues and future works for the proposed architecture to be verified experimentally and to be improved for more advanced applications listed as follows:

- The design with respect to the non-Newtonian characteristics of the fluids including blood such as modeling based on shear rate. Flowing effects and unstable movement of the donor particles should be accurately modeled with respect to the fluid mechanics to have the formulation depending on the viscosity.
- Utilization of the multiple donor lump spheres to improve the optical emission performance.
- In-vivo system design with the floating acceptor layers and the donor molecules with drastically different vibration properties.
- Extension to the flow rate measurement with the complex network of the donor and the acceptor pairs distributed to the medium with the corresponding algorithm.
- The proof of concept design of the microfluidic chip including the photodetection, acoustic excitation and the viscosity calculating circuit as an all-in-one POC unit.

## 9. Conclusions

A novel microfluidic viscosity monitoring system architecture is presented with an acousto-optical mechanism by exploiting recently introduced VFRET. It is completely mechanical and adaptable to different environments as a low complexity solution compared with the state-of-the-art. The donor molecules spread to the medium are acoustically vibrated resulting in VFRET with the nearby acceptor molecules. The collection of optical signals remotely provides the advantages of remote and wireless monitoring capability. Theoretical modeling and numerical simulation of the proposed monitoring system are provided with experimentally feasible parameters of the set-up. Finally, open issues and experimental challenges are discussed including the utilization for point-of-care and environmental monitoring applications such as water or blood viscosity monitoring for widespread usage.

## Figures and Tables

**Table 1:** Donor and acceptor material properties (F: Fluorescein, T: TAMRA).

Property	F	T	Property	F	T
Excitation wavelength (nm)	500	550	Fluorescence lifetime (ns)	4	5
Emission wavelength (nm)	515	577	pH dependency	Yes	No
Absorption coeff. (M^−1^ cm^−1^)	$7\times 10^{4}$	$9\times 10^{4}$	Phase forms of the molecules	dispersed	drop casted
Molecular weight (g/mol)	332.31	430.46	Concentration or weight	4 μM	$10^{-6}$ mg/μm^2^
Quantum yield (%)	95	68	Diameter of the particle (nm)	0.69±0.02	4.8±0.08

**Table 2:** Simulation Parameters.

Property	Value	Property	Value	Property	Value	Property	Value
$d_{0,max}$	20 nm	$d_{p}$	[1,500] μm	$N_{min}$	T/(12Δt)	$N_{MC}$	20
$f_{0}$	[10,300] Hz	$k_{A}$	1	$N_{T}$	1000	$N_{m}$	20
$R_{0}$	10 nm	$P_{f}$	[1,20] Pa	$E_{min}$	0.01	Δt	20 μs
$c_{f}$	1543 m/s	$\rho_{f}$	1 g/cm^3^	$\mu_{f}$	[0.5,5] (Pa ms)		
